# Moving beyond radiographic alignment: applying the Wald Principles in the adoption of robotic total knee arthroplasty

**DOI:** 10.1007/s00264-022-05411-3

**Published:** 2022-05-09

**Authors:** Jess H. Lonner, Graham S. Goh

**Affiliations:** Rothman Orthopaedic Institute at Thomas Jefferson University, 925 Chestnut St, Philadelphia, PA 19107 USA

**Keywords:** Knee arthroplasty, Robotic, Efficiency, Ergonomics, Artificial intelligence, Cost

## Abstract

The use of robotics in total knee arthroplasty (TKA) is growing at an exponential rate. Despite the improved accuracy and reproducibility of robotic-assisted TKA, consistent clinical benefits have yet to be determined, with most studies showing comparable functional outcomes and survivorship between robotic and conventional techniques. Given the success and durability of conventional TKA, measurable improvements in these outcomes with robotic assistance may be difficult to prove. Efforts to optimize component alignment within two degrees of neutral may be an attainable but misguided goal. Applying the “Wald Principles” of rationalization, it is possible that robotic technology may still prove beneficial, even when equivalent clinical outcomes as conventional methods, if we look beyond the obvious surrogate measures of success. Robotic systems may help to reduce inventory, streamline surgical trays, enhance workflows and surgical efficiency, optimize soft tissue balancing, improve surgeon ergonomics, and integrate artificial intelligence and machine learning algorithms into a broader digital ecosystem. This article explores these less obvious alternative benefits of robotic surgery in the field of TKA.

## Introduction

Unlike other surgical specialties, the use of robotic technology in total knee arthroplasty (TKA) is a relatively recent phenomenon [1]. For the most part, the contemporary field of robotics in orthopaedic surgery took root in unicompartmental knee arthroplasty (UKA) between 2007 and 2009 [2–6], but broader adoption has only occurred in the last five to seven years, as technologies have expanded to include TKA. This has been spurred by data confirming the safety of semi-autonomous systems, as well as improvements in navigation software, pricing, surgical efficiencies, and market access, with multiple robotic systems introduced into the market recently [2, 3, 5, 7]. The use of robotics in orthopaedic surgery is now growing at an exponential rate [8]. One statewide database reported that the utilization of robotics in arthroplasty increased from 16.2 to 29.2% of hospitals and 6.2 to 16.7% of surgeons between 2008 and 2015 [9]. It is anticipated that an updated analysis of the most recent five years will show an even greater use of robotic technology in TKA [10]. A recent survey of the membership of the American Association of Hip and Knee Surgeons (AAHKS) also found that 33% were using robotic assistance for TKA [11]. Analysts suggest that once robotic penetration in the arthroplasty market achieves a 35% level, orthopaedic surgeons and hospitals will routinely demand access to robotic technologies [12]– that threshold is quickly approaching.

While robotic-assisted systems were introduced with the prospect of enhancing surgical planning, individualizing component sizing and positioning, and quantifying soft tissue balancing, the ultimate goal is to improve functional outcomes and implant durability [2, 3, 13–16]. Indeed, robotic assistance has demonstrated measurable improvements in the precision of surface preparation and implant positioning in TKA compared to conventional techniques [13, 17–23]. In a meta-analysis of five prospective studies, Mannan et al. found that mechanical axis outliers occurred in only one of 181 (0.01%) robotic-assisted TKAs compared to 42 of 159 (26.4%) conventional TKAs [24]. Based on a recent AAHKS survey, improved precision may alone be enough impetus for 73% of surgeons to adopt robotic technology for TKA [11]. Despite the improved accuracy of robotic-assisted surgery, consistent clinical benefits of this technology have yet to be determined, with most studies showing comparable functional outcomes and survivorship between robotic and conventional techniques [17, 25–30]. In view of these contradictory reports, nearly one-third of arthroplasty specialists have expressed reluctance to adopt these technologies [11], and others have questioned the value of robotic assistance [31].

A paradigm shift is appropriate for how we perceive the role of robotic technologies vis-à-vis TKA. We may be thinking about robotics through too narrow a prism — if we use improved function and durability as explicit goals, robotic assistance may have a greater role for novice or low-volume surgeons, who may have difficulty achieving adequate precision *and* balance with conventional instrumentation [32], and for whom common errors from inexperience can be neutralized [14, 33, 34]. It is also possible that although some robotic systems are effective in optimizing both alignment and soft tissue balance, the relative importance of these capabilities may differ between procedures. For instance, in UKA, both precision of implant alignment and soft tissue balance are crucial for ensuring a successful outcome [35–37]; in TKA, on the other hand, recent data has suggested that variability in component alignment is well tolerated as long as the soft tissues are balanced [38–41].

How we think about robotic assistance in TKA invokes an account about Abraham Wald, a mathematician with whom the U.S. government consulted during World War II. Concerned about the state of fighter planes that were returning from combat missions with their fuselage and tails riddled with bullet holes, the military leadership sought a solution to reinforce the planes’ tails and fuselage without weighing them down and impairing their ability to fly. After contemplating the issue, Wald advised the group that their perception of the problem was misguided –– the planes that had been struck with bullets in the tails or fuselage were making it back safely –– they were not the problem. Rather, it was the planes struck in their noses and engines that were not returning, and thus it was the engines and noses of the planes that needed reinforcement and due consideration [42].

With this unconventional wisdom in mind, our efforts to optimize component alignment within two degrees of neutral may be an attainable but misguided goal. Minimizing errors in TKA makes intuitive sense, but this precision may not meaningfully impact implant durability or functional outcomes. In the spirit of what we will refer to as the “Wald Principles” of rationalization, it is possible that robotic technology may still prove beneficial, even with equivalent outcomes as conventional methods, if we look beyond the obvious surrogate measures of success. If by using robotic tools, we can reduce inventory, eliminate instruments and streamline surgical trays, improve surgical workflow and efficiency, quantify and optimize soft tissue balance, improve surgeon ergonomics and work effort, achieve net cost neutrality or even reduce costs through economies of scale, and integrate artificial intelligence (AI) and machine learning (ML) into a broader robotic TKA digital ecosystem, then its utility is supported. This article explores these less obvious alternative benefits of robotic surgery in the field of TKA.

## Reducing surgical inventory and improving operating room efficiency

As healthcare expenditures continue to grow at an unsustainable rate, hospitals and clinicians are under increasing pressure to deliver cost-efficient, high-quality care. Within this context, the operating room (OR) may represent a unique resource for hospitals to increase productivity and generate revenue, albeit at a high operating cost [43]. Consequently, the emphasis on OR efficiency has grown substantially [44]. Several factors have been shown to influence OR efficiency, including but not limited to the number of surgical instruments and trays, complexity of the surgical procedure, and implementation of complementary technology. The collateral benefit of robotic systems in reducing instrument tray burden can have a beneficial impact on reducing technician workload, instrument processing time and expenses, and OR setup time, although it has been challenging to perform robotic-assisted surgery with equivalent surgical time as conventional methods [45–49]. Furthermore, the cost savings from a reduction in instrument storage and sterilization may help to offset costs related to capital expenditures, per-case disposables and operational elements [50] associated with use of robotic technology in TKA [51].

Current semiautonomous and autonomous robotic systems rely on intraoperative navigation software to guide bone resection that may obviate the need for conventional alignment and cutting guides [52], hence decreasing the surgical inventory needed for each procedure. In addition, specifically for image-based systems, patient anatomy is mapped pre-operatively using advanced imaging to facilitate surgical planning prior to the procedure [53, 54]. This not only allows the surgeon to narrow down the range of implant sizes that may need to be available for the surgery, but also reduces the number of surgical instruments and trays required. This is a particularly valuable opportunity for cases performed in ambulatory surgical centers (ASCs), which have smaller capacity for instrument storage and sterilization. Case in point, in 2020, at least 23 ASCs in the USA added robotic platforms for TJA [55], and it is likely that these factors informed these acquisitions.

## Streamlining workflow inefficiencies in the surgical procedure

One of the intended goals of robotic technology for knee arthroplasty is to reduce surgical steps and enhance surgical efficiency [4]. Robotic systems require a period of training by surgical staff, and each system has a learning curve [48, 49, 56]. Nonetheless, setup of the robot and scrub table should not take longer than setting up a conventional procedure with standard instruments, and setup times should decrease as teams gain experience. Conventional TKA involves multiple surgical steps, with subtle variations based on surgeon and system philosophies, priorities and protocols. In contrast, even with the added time to register surface and limb landmarks in robotic-assisted TKA, 32% fewer steps may be necessary, again, with distinctions depending on robotic system used and surgeon-specific preferences in regard to workflow and trust in the system (e.g., need for verifications) (Table 1). Further, there are differences between systems in terms of the extent and numbers of registration points required for surface mapping in TKA. For example, one robotic system requires pre-operative planning from a 3-dimensional CT scan, followed by collection of 92 surface points intraoperatively [57]. A separate image-free robotic system requires continuous surface mapping of the entirety of the condylar surfaces [58]. A third robotic system, also image-free, has streamlined surface point collection to 17 points without compromising accuracy of preparation [15, 59]. Systems that predefine the outer margins of bone resection based on implant sizing may require freehand cutting of peripheral bone (beyond the implant size) or modification of the resection zone or plan virtually for resection of a larger implant, unless the implant extends to the margins of the knee [4, 24, 58, 60]. This is particularly germane with symmetric tibial components, which when positioned with appropriate rotation will almost routinely require further freehand resection of retained posteromedial bone due to asymmetry between the medial and lateral tibial hemiplateaus. On the other hand, systems that allow freehand resection through robotically positioned saw guides will not require override of system constraint [18, 25, 38, 61]. Each step in TKA — whether done with standard instruments or with robotic assistance — has a time element, and each surgeon has their individual preferences, algorithms, and efficiencies. Typical experience is that compared to conventional methods, after the learning curve period, robotic assistance requires a few more minutes up front to register landmarks and plan bone resections as well as ligament balance, but saves time in resection checks and recuts [33].

**Table 1:** Senior author bone preparation steps in conventional TKA and robotic TKA (excluding patellar resurfacing and implant trialing)

Steps	Conventional	Robotic
Insert tracking arrays		X
Register hip center		X
Register surface landmarks		X
Tibial resection		
Remove osteophytes	X	X
Stress soft tissues	X	X
Determination of tibial and femoral resection levels/orientations/slope, femoral AP translation, and implant sizes based on a combination of pre-resection surface mapping and intraoperative virtual soft tissue balance assessment		X
Set tibial guide, slide distal portion medial over center of tibial crest, medial portion over medial edge of tibial tubercle. Set coronal alignment	X	
Dial in slope (predetermined vs. match patient slope) with angel wing	X	
Put in 10/2 stylus to determine depth of resection	X	
Pin the guide in place	X	
Make saw cut through tibial guide	X	X
Remove wafer	X	X
Check coronal alignment with trial and drop rod	X	
Check coronal alignment using robotic validation tool		X
Femoral resection		
Set guide valgus angle	X	
Set guide resection depth	X	
Drill starting hole	X	
Adjust depth based on visual assessment, presence of large flexion contracture, etc. (− 2 mm for CR)	X	
Pin guide in place	X	
Remove rod	X	
Make saw cut through femoral guide	X	X
Confirm accuracy ^by visually assessment– recut if needed *using robotic validation tool (robot only)	X	X
Put 10 mm spacer block in extension space to confirm full extension; tension knee in extension, stressing ligaments medially and laterally, with valgus and varus stress, respectively	X	X
Adjustments in resection depths/orientations and/or soft tissue releases to guide quantified soft tissue balance medially and laterally in extension	X	X
Remove pins	X	
Tension knee at 90 degrees (*allow robotic system to autonomously determine femoral rotation to match extension)		X
Draw secondary rotational landmarks (Whiteside/TEA), especially for valgus knee	X	
Apply femoral sizing guide	X	
Set rotation, AP offset, size based on desired flexion gap	X	X
Drill alignment holes (*robot positions pin holes)	X	X
Impact 4-in-1 guide in place, secure in place	X	X
Cut through slots	X	X
Final confirmation after trialing to confirm position before final implantation if desired	X	X
Make two bone plugs from anterior chamfer cut to plug femoral canal	X	

^For conventional TKA only

^*^For robotic TKA only

## Intra-operative customization to impact patient satisfaction and quality of life

One important advantage of robotic-assisted TKA is the high intra-operative adaptability and customization based on patient-specific knee morphology and soft tissue balance. Using dynamic real-time intra-operative assessment of the flexion and extension gaps, quantified kinematic bone resections or additional soft tissue releases can be made [59, 62]. This high level of individualization obviates the need for patient-specific instrumentation and customized implants [60], and facilitates quantified intra-operative adjustments. Given the growing interest in applying the principles of restricted kinematic alignment in TKA, robotic assistance provides the ideal mechanism for responsibly modulating alignment and balance [62].

Compared to conventional instrumentation [63], the improved intra-compartmental ligament balance provided by several semi-autonomous robotic systems may ultimately lead to better patient-reported knee function and satisfaction compared to manual TKA, as we are beginning to observe [61, 64, 65]. This is a contradistinction from the results of robotic systems that do not include a protocol for soft tissue balancing [27, 28]. One study found that patients undergoing robotic-assisted TKA with a system that utilizes a soft tissue balancing algorithm had significantly greater satisfaction compared to a matched group of patients undergoing conventional TKA at one year (94 vs. 82%) [66]. Furthermore, Knee Society scores were significantly higher in the robotic group, suggesting a potential benefit of the soft tissue balancing algorithm. Another study found that a robotic system that integrated real-time intra-operative alignment and gap balancing information yielded greater improvements in all subscores, as well as sports and recreation outcomes measures at two years compared to previously published registry data [67]. Finally, emerging data suggests that patients experience significantly less audible noise, and symptoms such as grinding, popping, or clicking, in robotic-assisted TKAs compared to conventional TKAs, and these patients are more likely to achieve the patient acceptable symptom state (PASS).

## Less post-operative pain and lower opioid consumption

Another possible advantage of robotic-assisted TKA is decreased early post-operative pain and reduced opioid requirements after surgery. Bhimani et al. found that patients who underwent robotic-assisted TKA had lower pain scores at two and six weeks post-operatively [68]. More importantly, these patients required significantly less morphine equivalents per day compared to patients who underwent conventional TKA, and a significantly greater percentage was opioid-free by six weeks (71 vs. 57%). While the exact mechanism of pain relief is unknown, it is posited that this may be due to better quantification of soft tissue balance or decreased iatrogenic bone and soft tissue trauma in robotic-assisted cases [16, 61, 69]. Further studies are necessary to determine the generalizability of this data depending on robotic systems, surgical techniques and peri-operative protocols, and to determine whether the difference in pain relief can be sustained in the long-term.

Improved ergonomics, reduced physiologic strain.

### Reducing work-related injuries

The incidence of musculoskeletal strain and injury among orthopedic surgeons has been reported to reach 67%, for which 27–31% required time off from work [70–72]. Arthroplasty surgeons are often at increased risk for musculoskeletal injury [73, 74], particularly given the rising mean age of orthopaedic surgeons in the USA [75]. Moreover, approximately one-third of surgeons may experience pain levels that exceed the threshold which would permit the use of prescription narcotics after their operating day [76]. Despite the high prevalence of musculoskeletal complaints among surgeons performing joint replacement surgery, there is a paucity of research on the risk factors for work-related musculoskeletal injuries and how to optimize intra-operative ergonomics in order to mitigate them. Like the robotic systems that were first developed to help surgeons tackle ergonomically challenging laparoscopic tasks [1], it is possible that contemporary robotic technology could reduce ergonomic strain and physiologic stress during TKA [77, 78]. In a recent cadaveric study, surgeons who performed conventional TKA spent 15% more time in a nonneutral cervical spine position compared to those who performed robotic-assisted TKA cases [77]. Similarly, a clinical study of 40 TKAs performed with either robotic surgical assistance or conventional instrumentation reported a reduction in energy expenditure, surgeon stress, and lumbar strain with robotics, despite longer operative times during the initial robotic cases [78]. The streamlining and elimination of surgical steps, coupled with less time in demanding positions, demonstrate the utility of robotic systems in substituting laborious tasks with more ergonomic ones. Considering the growing volume of joint replacement procedures and the increasing recognition of musculoskeletal injuries among arthroplasty surgeons, determining whether enabling technologies can provide additional value by preserving orthopaedic surgeons’ health and safety remains paramount.

### Robotics for the aging surgeon

As the global population ages due to increasing life expectancies, it important to acknowledge that the surgical workforce is no exception. According to the Association of American Medical Colleges Physician Specialty Data Report, 44% of 103,032 practicing surgeons in the USA were above the age of 55 in 2017 [79]. The mean age of orthopaedic surgeons increased from 50.7 to 56.5 years between 2008 and 2018 [75]. This remains an important consideration, since the wealth of knowledge and experience that older surgeons can offer needs to be weighed against the potential compromise in surgical performance due to an inexorable decline in physical and cognitive functioning that accompanies aging. In addition to improved ergonomics, robotic assistance may improve surgical precision for older surgeons facing psychomotor difficulties and allow them to maintain a high degree of accuracy during the procedure, although this requires further study. Navigation software and intrinsic AI algorithms can also aid in surgical planning and ease the cognitive burden. Robotic systems not only enhance productivity for surgeons, but may also help to maintain high performance standards and ensure the longevity of a surgical career for aging surgeons who wish to continue their practice.

## Reduced peri-operative costs

The cost of robotics in TKA is perhaps the greatest barrier to more widespread adoption [1]. As the prevalence of robotic-assisted surgery is expected to increase in the near future [8], understanding the cost-effectiveness of robotic assistance in arthroplasty is crucial. In a Markov decision analysis, Moschetti et al. determined that robotic-assisted UKA was cost-effective when case volume exceeded 94 cases per year, two year failure rate was below 1.2%, and total system costs were less than $1.426 million [80]. However, the analysis was modelled based on an older robotic acquisition model which required high capital outlay of $1.362 million and preoperative CT scans of $247 per scan. Other authors have predicted that incremental increases in the number of robotic TKA procedures could lead to a return on investment in approximately two years with a single application [81]. More recent studies examining the impact of robotic assistance on the entire 90-day episode of care have similarly noted that higher intra-operative costs (capital costs of the robot, maintenance fees, and robot-specific disposables) were offset by lower post-operative 90-day episode-of-care costs (reduced instrument processing fees, shorter length of stay, and reduced opioid requirements) [82, 83]. Cool et al. also demonstrated that overall 90-day episode-of-care costs for robotic TKA patients were 11% lower than that for manual TKA patients ($18,568 vs. $20,960) [84]. Post-acute savings were attributed to lower non-home discharges, fewer skilled nursing facility admissions and less emergency room visits in the 90-day period, and these findings were echoed by Emara et al. recently [8].

## Artificial intelligence and machine learning

AI has proven its usefulness in healthcare systems because of its ability to handle and optimize large, complex datasets [85], demonstrating the proficiency of modern computing in tracking and analyzing multiple variables in a time-efficient manner. In the context of orthopaedic surgery, machine learning methods have been increasingly used to predict peri-operative complications [86], blood transfusions [87], length of stay [88], opioid use [89], functional outcomes [90], patient satisfaction [91] and early revision [92] following TJA. Perhaps the greatest area of untapped potential for robotics is the possibility of integrating AI to individualize intra-operative decision-making with regard to soft tissue balance and component positioning. This powerful tool has already been integrated into some robotic systems, including one which uses AI to determine femoral component rotation and sizing based on intra-operative assessment of the medial and lateral extension gap balance [59]. In its capacity, AI can thus simplify the complexities of managing numerous interrelated and constantly changing variables and data points (e.g., soft tissue balancing, resection depth, alignment etc.).

As data is synthesized within a digital ecosystem that includes patient-specific information collected intra-operatively using robotic technology as well as peri-operatively using smartphone or wearable technology, AI algorithms will be refined to guide surgical decisions to drive efficiencies and ultimately influence outcomes [93]. These applications may prove to be even more advantageous compared to the obvious benefits of improved alignment accuracy currently offered by robotic systems. As the volume of intra-operative data collected increases exponentially with robotic technologies [9], this will indubitably improve our understanding of joint kinematics and inspire the development of newer surgical software to optimize outcomes. Current robotic systems are still at the stage of collecting intra-operative data without knowing the best way of utilizing it. With the advancement of AI and its applications in big data, newer robotic systems integrating dynamic data insights and machine learning could improve surgical decision-making by doing what it does best: collecting and analyzing a vast amount of data at unimaginable speeds and presenting the most clinically relevant information to the surgeon. By doing so, it is our expectation that highly individualized, reproducible, and meaningful results may be achieved for the entire episode of care of patients undergoing TKA. The next generation of orthopaedic surgeons will likely capitalize on these evolving data intelligence capabilities to allow mass customization of TKA.

## Conclusion

The proliferation of robotic systems in orthopaedic surgery over the past decade is an anticipated progression of the digital revolution which began in the late twentieth century [94]. Although the cost of robotic surgery is still relatively high, greater competition within the industry, improved manufacturing productivity, and non-capital based acquisition strategies are ushering in a period of substantial cost reduction [3]. While improved component alignment and positioning are used as proxy determinants of the benefit of robotic technology, it is not definitive that these improvements alone can influence clinical outcomes and survivorship in TKA. Nonetheless, even if outcomes are equivalent despite the improvements achieved with robotic assistance, we can apply the “Wald Principle” to argue that there are alternative benefits of robotics for TKA. As surgeons continue to debate the utility of robotics in healthcare, it is important for all stakeholders to consider these underappreciated benefits of robotics in surgery, especially in the context of growing surgical volumes, an aging population of arthroplasty surgeons and an increasingly digitalized world. Transition to ASCs and orthopaedic specialty facilities with smaller space for storage and sterilization, quantification of soft tissue balance and implant positioning, and optimized ergonomics and physiologic stress are worthy considerations in robotic TKA, provided that costs and surgical efficiencies are managed. Further, integration of AI will likely improve surgical workflow and may prove to have a profound impact on patient outcomes. Ultimately, the exact role and potential of robotics may not yet be clear. To paraphrase the author Yuval Noah Harari [95], many emerging technologies are advancing faster than our understanding of them — this may very well be the case with robotics in TKA.

## Data Availability

Not applicable.
